# The Mechanism of Physical Activity-induced Amelioration of Parkinson’s Disease: A Narrative Review

**DOI:** 10.14336/AD.2020.0407

**Published:** 2021-02-01

**Authors:** Piotr Gronek, Aline Nogueira Haas, Wojciech Czarny, Robert Podstawski, Marcela do Santos Delabary, Cain CT Clark, Michał Boraczyński, Maria Tarnas, Paulina Wycichowska, Mariola Pawlaczyk, Joanna Gronek

**Affiliations:** ^1^Laboratory of Genetics, Department of Health Sciences, Poznań University of Physical Education, Poznań, Poland.; ^2^School of Physical Education, Physiotherapy and Dance, Federal University of Rio Grande do Sul, Porto Alegre, Brazil.; ^3^Department of Human Sciences, University of Rzeszów, Poland.; ^4^Department of Tourism, Recreation and Ecology, University of Warmia and Mazury in Olsztyn, Olsztyn, Poland.; ^5^Faculty of Health and Life Sciences, Coventry University, Coventry, United Kingdom.; ^6^Public Health, University of Warmia and Mazury Olsztyn, Poland.; ^7^Department of Geriatric Medicine and Gerontology, Poznań University of Medical Sciences, Poznań, Poland.

**Keywords:** Parkinson disease, physical activity, motor symptoms, cognitive networks, mind-body interventions

## Abstract

Physical activity, together with its ameliorative effects on Parkinson’s disease (PD) symptoms, remains a relatively unappreciated factor which may be beneficial for the treatment outcome. Contemporary evidence supports the positive effects of non-pharmacological approaches to PD symptom management, in particular the effects of the exercise on both, motor and non-motor symptoms. The aim of the study was to review the mechanisms of exercise-induced amelioration of PD symptoms. Methods: Electronic databases (PubMed, Web of Science and Google Scholar) were searched using the following key words: “Parkinson and physical activity” OR “Parkinson disease and exercise” OR “Parkinson disease and lifestyle factors” OR “Parkinson disease and longevity”. A total of 97 studies which investigated PD genetics and various forms of exercise and their etiologic impact on PD were reviewed. The studies were subdivided into four topic groups: 1) genetics of PD, 2) exercise and the brain, 3) physical activity and PD, 4) mind-body interventions, and discussed accordingly. Adequate levels of physical activity are associated with higher quality of life in PD patients. Physical activity may have protective and stimulatory effects for better functional efficiency in higher-level cognitive networks. It can also improve balance and motor functions by improving muscle strength. Given the etiologic evidence of the beneficial effects of physical activity on PD, albeit tentative, a concerted effort to elucidate the processes and outcomes of physical activity on ameliorating symptoms of PD must be undertaken.

The year 2017 marked the 200-year anniversary of the initial diagnosis of Parkinson’s Disease (PD) described by James Parkinson [1]. However, despite remarkable progress in our understanding of PD etiology and pathogenesis [2], certain key questions remain unresolved. PD is also known as older person’s disease, although a growing percentage of patients have been diagnosed with early-onset parkinsonism subdivided into cases with onset before age 21 years (juvenile parkinsonism) and those with onset at or above age 21 years (young onset Parkinson disease [YOPD]) [3]. PD is the second most common age-related neurological disorder, marked by degeneration and the loss of predominantly dopaminergic neurons in the substantia nigra [4], associated with intracellular, insoluble α-synuclein (α-Syn) aggregates, localized to Lewy bodies and within neuronal processes, termed Lewy neurites. PD involves both, motor and non-motor symptoms (NMS), affecting 7-10 million people around the world [5]. It is estimated that 2,802 per 100,000 persons in North America, Europe and Australia are affected by PD [6], with the main symptoms manifesting as bradykinesia (slow movement), tremor, rigidity (increased muscle tone), postural instability, altered gait pattern, freezing of gait and motor coordination deficits [7, 8] and shortened stride length [9].

The visible symptoms are connected with motor control and mobility in general, however most patients are also affected by NMS, including bladder dysfunction [10], pain [11], insomnia [12], cognitive impairment and dementia [13], depression and anxiety [14], osteoporosis [15], constipation [16], apathy [17], and general fatigue [18], with the general effect of fatigue on human performance in older adults remaining the topic of debate [19].

Contemporary evidence supports the positive effects of non-pharmacological approaches to PD symptom management, in particular, the effect of the exercise on both, motor and non-motor symptoms [20]. Exercise is speculated to be underutilized due to various reasons, chief among them limited knowledge on its therapeutic potential [21]. Unfortunately, many patients with Parkinson’s disease lead a sedentary lifestyle due to their combined physical limitations and mental changes [22]. Therefore, in 2010 a multifaceted and individually tailored training programme to improve PA in sedentary patients with PD was developed (ParkFit) [23]. The implementation of this programme led to greater participation in specific elements of physical activity (PA) and showed an improved physical fitness among ParkFit patients. While pharmacotherapy is essential in PD therapy, the combination of medication and PA may constitute a conceivable way to minimize various disease-induced limitations [24, 25].

A number of questions concerning PA and its ameliorative effects on PD symptoms (such as volume, intensity, type of PA, including the optimal intensity and volume per week, the timing of the exercise intervention relative to disease onset, and likewise type of exercise necessary for maximal neuroprotection and neurorestoration) have not yet been precisely defined and researchers lack consensus on the matter [26].

Given the potential for PA to alleviate symptoms of PD, and in the absence of the consensus, the aim of the study was to review the mechanisms of exercise-induced amelioration of PD symptoms.

## MATERIALS AND METHODS

### Search strategy

The search focused on PD in relation to PA. Electronic databases (PubMed, Web of Science and Google Scholar) were searched using the following keywords: “Parkinson and physical activity” OR “Parkinson disease and exercise” OR “Parkinson disease and lifestyle factors” OR “Parkinson disease and longevity”.

### Study Characteristics

Multiple searches in each of the selected databases and additional searches for relevant references and citations were conducted. The eligibility criteria in the selection process were used to identify experimental or observational studies on PD in relation to PA or exercise at any age. Only articles written in English between January 2000 and August 2017 were considered. Two of the authors assessed all titles and abstracts and all full-text articles. The decision to accept or reject a paper was made by the first and the second author. A third, independent reviewer helped achieve a consensus, if needed.

## RESULTS AND DISCUSSION

**Figure 1.**
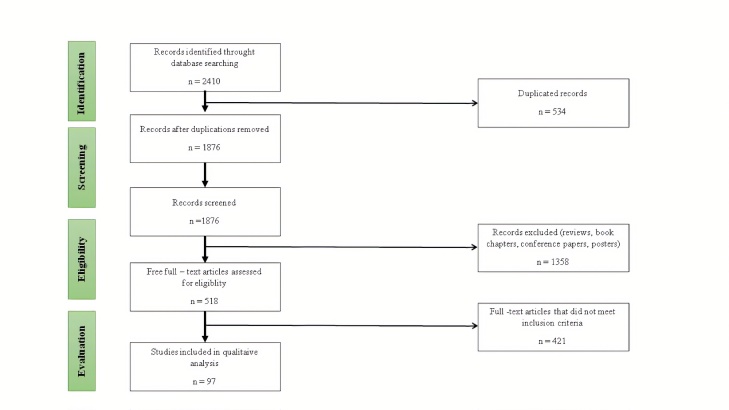
Flow chart of the study selection procedures.

Out of the 2410 articles identified in the databases, 1876 studies remained after removal of the duplicates. After reading the titles and abstracts, 421 articles were excluded for not having PD patients as participants. The 1455 studies selected for phase 1 were fully read, and 1358 studies failed to meet the eligibility criteria and were excluded. Overall, 97 studies, compliant with the eligibility criteria of this research, were included. Figure 1 shows the flowchart of the studies included in this review (Fig. 1).

Following screening and detailed assessment, the included studies were subdivided into four topics: 1) genetics of PD, 2) exercise and the brain, 3) PA and PD, 4) mind-body interventions, and discussed accordingly.

### Genetics of PD

Although the environmental factors play an essential role in the development of PD [27,28]**, recent studies have suggested that a number of genes influence susceptibility to this neurodegenerative disorder, including the α-Syn gene [29,30], autosomal recessive juvenile parkinsonism (AR-JP) [31], PTEN-induced putative kinase 1 (PINK1) [32], DJ-1 gene [33], and neuronal P-type ATPase gene [34].

In a large genome-wide association study (GWAS) of PD, with over 3.400 cases and 29.000 controls (the largest single PD GWAS cohort to date), and after controlling for age, sex, genotyping platform, and five principal components, Do *et al*., identified two novel regions-SCARB2 and SREBF1/RAI1-and replicated six previously reported regions-LRRK2, SNCA, GBA, MAPT, MCCC1/LAMP3, GAK and replicated a total of twenty previously described associations of genetic factors with PD [35].

Summarizing, it is estimated that genetic factors may contribute to at least 25% of the total variation in susceptibility of the variation to PD liability, of which currently discovered factors only explain 6%-7% [35], so the remaining 93% are still ambiguous.

### Exercise and brain

Certain regions of the brain routinely increase their activity while performing attention-demanding cognitive tasks, whereas others habitually decrease their activity [36]. The frontoparietal (FP) network, Default Mode Network (DMN) and the fronto-executive network (FE) act on the basis of the efficient communication between the frontal cortex and the rest of the brain. Aging negatively affects these networks and, as a consequence, specific dysfunctions of the brain networks appear [37]. Daydreaming, mind-wandering, thinking, planning for the future and autobiographical memory intensively activate the DMN [38,39]. This network has been relatively well-described, and it is known to include brain structures such as parahippocampal and hippocampal, posterior cingulate, ventral and superior frontal medial cortices, and bilateral lateral occipital, middle frontal, and middle temporal cortices [36,38]. Decreased DMN activity is associated with deteriorating working memory [40] and performance of executive function tasks in older adults [41,42]. The important question remains whether exercise can ameliorate the dysfunction of the aging brain. Voss *et al.*, [37] used functional magnetic resonance imaging (fMRI) to examine low-frequency (0.008 < *f* < 0.08 Hz) coherence of cognitively relevant and sensory brain networks in older adults (1-year intervention trial, walking), and compared the effects of nonaerobic and aerobic fitness training on brain function and cognition. The authors highlighted that aerobic training improved the aging brain’s resting functional efficiency in higher-level cognitive networks. Attributed to PA, functional connectivity within the DMN and the Frontal Executive Network significantly increased, ameliorating, in particular brain dysfunction in the aging subjects.

Exercise is an essential impulse for various cells (e.g. myocytes) to upregulate the expression of insulin-like growth factor type 1 (IGF-1) [43,44], brain neurons to increase production of neurotrophic factors such as brain-derived neurotrophic factor (BDNF) [45,46] and limiting for neuroinflammation [47,48]. Moreover, exercise regulates plasticity in the hippocampus and the cortex [49], CA1 and entorhinal cortex [50,51], and increases fine discrimination [52]. The effect of PA, especially on the nervous system, results in increased molecular adaptations in neuronal function [53]. However, many of the mechanisms by which exercise exerts its effects in the brain remain largely unknown.

**Figure 2.**
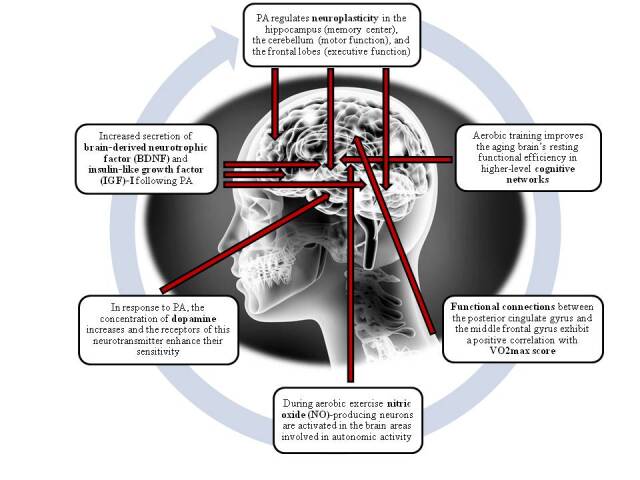
Summary of the main pathways effect of aerobic exercise on the nervous system.

Figure 2 shows the summary of the main pathways effect of aerobic exercise on the nervous system (Fig. 2).

Exercise - Human movements and physical activities involving large muscle groups, rather than highly specific, relatively non-taxing movements of small muscle groups. Exercise includes dance, callisthenics, games, and more formal activities such as jogging, swimming, and running [54]

Studies on animal models have shown that aerobic exercise can, putatively, reverse the decline in neurogenesis and memory function [55,56,57], exert positive influence on cognition (especially hippocampus-supported learning and memory) [58], and increase the volume of grey matter in the region of the right EC. Moreover, Duchesne *et al*., [59] suggested that aerobic exercise training can reduce the symptoms in the early stages of PD, not only eliciting improved physical fitness, but also motor learning capacity, which is useful in habitual activities through increased plasticity in motor-related structures [59]. The effects of physical activity, both endurance and weight training, on brain function have been discussed in detail in a review paper by Di Liergo *et al*. [60].

### Physical activity in Parkinson’s Disease

During the analyzed period, 2410 articles on the health benefits of physical activity in PD were published, including 379 Randomized Clinical Trials (RCTs). Besides pharmacotherapy, a number of complementary approaches have become available; a) aerobic and anaerobic exercise, b) complementary and alternative medicine (CAM), with mind-body medicine being the most commonly used form in the USA [61], and c) dance, as the transitive approach including both, PA and some CAM elements. Sufficient exercise improves aerobic and anaerobic fitness, indirectly eliciting general health benefits, and improves motor habitual behaviors, especially in PD patients. Whilst aerobic training is generally operationalized in a standardized manner, anaerobic exercise (i.e. 10-m maximal walking speed tests or peak power for leg press) is often used interchangeably with resistance training or with strength training, and therefore warrants careful interpretation. Resistance training is generally concerned with lower loads and more repetitions than the general strength training, which is usually a combination of the two types of hypertrophy: i.e. myofibrillar hypertrophy (increased myofibril size), where contractions against 80% to 90% of the maximal voluntary contraction (MVC) occur and sarcoplasmic hypertrophy (increased in myocytes glycogen storage). In summary, strength exercise, power-strength exercise, strength-endurance exercise are numerous terminologies associated with training activity, and each must be carefully interpreted and operationalized cautiously, per individual capability.

### Aerobic exercise

Concurrent motor impairment in PD [62,63,64,65] and ways of eliciting improvement in habitual activities, motor performance, ambulation and the overall functional independence [66], remain the challenge of the contemporary physical therapy modalities. Continuous exercise training for 12 weeks in mouse model of Parkinson’s disease (MPD) showed that, in spite of the drastic loss of dopaminergic neurons and depletion of dopamine in the severe chronic MPD, endurance exercise training effectively reverses the Parkinson's like behavioral deficits related to regular movement, balance and gait performance. Unfortunately, long-term exercise training did not reverse the habitual learning deficit in chronic MPD [67]. Given the potential of the aerobic exercise to elicit such positive adaptations, its use should be advocated. Notwithstanding, more randomized controlled trials in humans are required to obtain any consensus.

### Muscle strength

Analysis of the studies which investigated muscle strength is more complicated than studying aerobic training (mostly walking, cycling) since a plethora of different modalities and terminologies are available, including the maximal voluntary contraction and the rate of force development [68], peak muscle power [69], one-hand grip, and pinch strength [70], muscle isometric contraction [26,69,70,71,72,73], and other physical tests and no instrumental evaluation [26]. A systematic review of the effectiveness of strength training, performed against a different resistance from body weight, in improving motor and non-motor symptoms in patients with PD recently concluded that strength training appears to be a suitable PA to improve a number of physical parameters, especially maximal voluntary contraction, the rate of force development, balance, reaction time, gait speed and many others. Moreover, quality of life parameters assessed by different questionnaires: UPDRS-II, Parkinson’s Disease Questionnaire, Parkinson’s Disease Quality of Life Scale, and Beck Depression Inventory were also improved in PD subjects [74,75].

Investigating the effects of physiotherapy with concurrent strength training (ST) and aerobic training (AT) on motor symptoms, functional capacity, and electroencephalographic (EEG) activity in PD patients, Carvalho *et al*., [26] observed that aerobic and strength training, controlled by parameters of intensity, duration, and frequency, showed a greater improvement than conventional physical therapy as far as symptoms and cortical activity of PD patients were concerned: strength training - improvement by 27.5% (effect size [ES]=1.25, confidence interval [CI]=-0.11, 2.25), aerobic training - by 35% (ES=1.34, CI=-0.16, 2.58), physiotherapy group - by 2.9% (ES=0.07, CI=-0.85, 0.99) [26].

### Mind-body interventions

Mind-body therapies which can be applied in PD patients include systems associated with kinesthesia, such as Yoga [76], Tai-chi [77], or Qigong (part of the Tai-chi system) [78]. In this area, dance has emerged as an important tool in the complementary to pharmacotherapy treatment of PD patients.

Other possibilities include systems which are not associated with movement, such as mindfulness-based cognitive therapy (MBCT) [79], meditation [80], breathing techniques [81], hypnosis [82,83] and biofeedback [84].

### Yoga

During the analyzed period, 32 articles were published, including 3 RCTs and 29 systematic reviews on the health benefits of Yoga in PD. Recently, Yoga practice has increasingly been used for enhancing well-being, stress reduction, and improving coping with chronic diseases of aging, and various neurodegenerative disorders such as PD [85,86,87]. In Poland 80% of all Yoga practitioners are female and 12.5% have Ph.D. or higher education, thus it could be stated that this kind of PA is popular among well-educated women [88].

Analyzing the use of Yoga only in the context of PD, a number of benefits come to mind. Firstly, the use of inexpensive interventions which combine movement, chanting, with breath practices and meditation, can help achieve some therapeutic goals via non-pharmacological stress reduction, brain enhancement exercise, lifestyle changes [80], and appears to improve mood, coping with chronic stress, and provides relief of chronic pain [89].

Secondly, Yoga exercises are less strenuous as compared to many others so they can be used in older adults with physical limitations and many of the practices may be adapted to a sitting or horizontal position. Finally, Yogic activity, including meditation, is a safe, non-pharmacologic approach, thus reducing polypharmacy. PD progression is the consequence of the loss of predominantly dopaminergic neurons in the substantia nigra, and the concentration of dopamine decrease [4], Kjaer *et al*., [90], using 11C-raclopride PET, *in vivo*, demonstrated a 65% increase in endogenous dopamine release in the ventral striatum and conscious experience during Yoga Nidra meditation, suggesting that while meditating, a suppression of cortico-striatal glutamatergic transmission occurs [90].

### Tai-chi and Qigong

The reviewing process identified 63 articles, including 10 RCTs and 53 systematic reviews on the health benefits of Tai-chi and Qigong in PD. Tai-chi, also known as T'ai chi ch'uan or Taiijiquan, is a meditative martial art which can be considered as a mind-body exercise [91]. Since Qigong is a part of the Tai-chi system, these techniques may be analyzed together.

Tai-chi consists of a series of dance-like movements flowing smoothly from one position to another, emphasizing weight transfer during kinesthesia of the body. All transitions are integrated with breathing and finally linked in a continuous sequence [92]. All movements are characterized by a low-impact and simple flow, which is the fundamental reason for their acceptance by the older people.

Parallel to motor function deterioration, which is typical for PD, falling, which is the result of muscles weakness, balance problems and freezes, is one of the most common problems in Parkinson’s patients [93, 94]. Following Qigong scheduled exercises, 5 times per week for 60 min. each time (10 min. for warm-up, 40 min. for the exercise, and 10 min. for cooldown), showed significant improvement in muscle hardness, timed “up and go”, balance, and hand-eye coordination (the turn-over-jars test) [78]. Moreover, Tai-chi has been shown to significantly improve balance [95], strength of the lower limbs [96], and flexibility [97].

In summary, Tai-chi and Qigong might be considered complementary to medications. The combined approach could achieve superior results; however, it should be emphasized that their role is not to cure but help maintain as much normal function as possible in the affected individuals.

### Dance in PD

There is strong evidence to suggest that exercise programs for patients with PD can improve mobility [98], balance [99], strength [100], and consequently, quality of life [101].

In the last 10 years, several studies have sought to investigate the effects of dance on people with Parkinson’s. As part of the review process, 19 RCTs on the health benefits of dance in PD were identified. The RCTs generally focused on coordination, balance, functional mobility, spatial cognition in PD. In addition to mind-body medical approaches to exercise such as Yoga, Tai Chi, dance has also been studied as an appropriate and useful intervention [102].

Dance is often associated with ballroom dancing, often out of reach for the ordinary, physically non-active person, however there are numerous types and styles of dances which are studied in the context of older adults, including ballroom [103, 104], various types of Latin [105, 106] traditional [107, 108, 109], and contemporary dance [110, 111].

Music and body movement are the common features of all styles and types of dance. Dance may be a relevant form of physical activity, especially in PD and older adults, due to the fact that the range of intensity, and flexibility may be adapted to individuals. Dancing may be a popular mode of leisure time for older people. However, it is still alarming that physical activity of 60% Americans over the age of 65 is much lower than recommended [112]. Additionally, it has been shown that dancing is associated with improvement in mobility [103], balance [113] and quality of life [109].

Gait difficulties observed in PD patients concern forward walking, but also backward walking, such as stepping back from a curb as a swiftly moving bus passes, as well as in older people, whose walking is characterized by lesser cadence, increased double support time, and shorter stride length and swing phase [114]. Also, Hackney and Earhart [115, 116] observed that during backward walking, gait speed and stride length in those individuals are reduced as compared to healthy controls.

Most of the RCTs in the area of dance for Parkinson’s propose tango as an intervention. Tango is defined by many short steps, alternately smooth and sharp, and for this reason it could be an interesting and promising therapy for improving gait, balance, and mobility, while also reducing disease severity in PD [113, 116].

### Clinical significance and implications

Recent RCTs have highlighted a possible association between PA or exercise and PD therapy. Exercise training, in early-stage PD, can also promote better motor learning capacity through increased plasticity in motor-related structures [59]. Carvalho *et al*., [26] found that combining classical physiotherapy with concurrent strength exercise and aerobic exercise - controlled by parameters of intensity, duration, and frequency, on motor symptoms, functional capacity, and EEG activity in PD patients the aerobic and strength training - resulted in greater improvement than conventional physical therapy as far as symptoms and cortical activity of PD patients were concerned [26].

The crucial accidence concerned falling, which is a common problem in PD, with significant risk of body injury. Resistance exercise, Tai chi, Yoga, and dance can improve mobility, balance, and strength, especially of the lower limbs, and by strengthening the muscles and improving balance, decrease falls and, as a consequence, improve quality of life among PD patients [101].

Yoga, being less strenuous as compared to many other exercises, can be used by older adults with physical limitations and may be adapted to a sitting or horizontal position.

### Conclusions

It is safe to conclude that the presented studies provide compelling evidence that regular exercise could improve muscle strength, balance and motor functions in PD patients. Further research is necessary to determine whether physical activity may have protective and stimulatory effects for better functional efficiency in higher-level cognitive networks.
